# Speed-adaptive control of functional electrical stimulation for dropfoot correction

**DOI:** 10.1186/s12984-018-0448-x

**Published:** 2018-11-06

**Authors:** Guangtao Chen, Le Ma, Rong Song, Le Li, Xiaoyun Wang, Kaiyu Tong

**Affiliations:** 1 Key Laboratory of Sensing Technology and Biomedical Instrument of Guangdong Province, School of Engineering, Sun Yat-sen University, Guangzhou, Guang Dong China; 2 Department of Rehabilitation Medicine, Sun Yat-sen First Affiliated Hospital, Sun Yat-sen University, Guangzhou, China; 3 Guangdong Work Injury Rehabilitation Center, Guangzhou, China; 4 Department of Biomedical Engineering, the Chinese University of Hong Kong, Hong Kong, China

**Keywords:** Electromyography, Walking speed, Dropfoot, Functional electrical stimulation

## Abstract

**Background:**

Functional electrical stimulation is an important therapy technique for dropfoot correction. In order to achieve natural control, the parameter setting of FES should be associated with the activation of the tibialis anterior.

**Methods:**

This study recruited nine healthy subjects and investigated the relations of walking speed with the onset timing and duration of tibialis anterior activation. Linear models were built for the walking speed with respect to these two parameters. Based on these models, the speed-adaptive onset timing and duration were applied in FES-assisted walking for nine healthy subjects and ten subjects with dropfoot. The kinematic performance of FES-assisted walking triggered by speed-adaptive stimulation were compared with those triggered by the heel-off event, and no-stimulation walking at different walking speeds.

**Results:**

Higher ankle dorsiflexion angle was observed in heel-off stimulation and speed-adaptive stimulation conditions than that in no-stimulation walking condition at all the speeds. For subjects with stroke, the ankle plantarflexion angle in speed-adaptive stimulation condition was similar to that in no-stimulation walking condition, and it was significant larger than that in heel-off stimulation condition at all speeds.

**Conclusions:**

The improvement in ankle dorsiflexion without worsening ankle plantarflexion in speed-adaptive stimulation condition could be attributed to the appropriate stimulation timing and duration. These results provide evidence that the proposed stimulation system with speed-related parameters is more physiologically appropriate in dropfoot correction, and it may have great potential value in future clinical applications.

**Trial registration:**

Medical Ethics Committee of Guangdong Work Injury Rehabilitation Center, AF/SC-07/2016.22. Registered 26 May 2016.

## Background

About three quarters of stroke survivors experience different levels of brain dysfunction and movement disorder [1], which result in lower living quality and limited ability in social activities [2]. Of these subjects, 20% suffer from impaired motor function in the lower extremities. One of such impairments is dropfoot, which is characterized by poor ankle dorsiflexion during the swing phase and an inability to achieve heel strike at the initial contact [3, 4]. Abnormal gaits such as circumduction gait and abnormal foot clearance on the affected side are often found as a method of compensating for excessive hip abduction and pelvis elevation on the unaffected side [5]. This results in gait asymmetry and slow walking speed [6].

Functional electrical stimulation was a representative intervention to correct dropfoot and Liberson et al. first introduced functional electrical stimulation (FES) to correct dropfoot for chronic hemiplegic subjects in the 1960s [7]. An electrical charge is delivered via a pair of electrodes to activate the tibialis anterior (TA), which results in ankle dorsiflexion. Yan et al. applied two dual-channel stimulators to the quadriceps, hamstring, gastrocnemius, and TA to recover motor function of the lower extremities in an early stage after stroke [8]. The stimulation was followed by a predetermined sequence of muscle activations that mimic a healthy gait cycle [9]. The duration of stimulation was five seconds in Yan et al.’s study. However, subjects with different severities of impairment might have different walking speeds [10], which means that a fixed stimulation duration might not be able to account for different walking patterns.

Liberson et al. used the heel-off event detected by a footswitch to trigger the stimulation [7]. However, the reliability of the footswitch controller was significantly reduced when subjects who dragged their feet during walking encountered a slope or an obstacle [11]. Bhadra et al. proposed a manual switch to trigger stimulation as a walking aid for subjects with spinal cord injury (SCI) [12]. However, manual control may distract subjects from maintaining balance and lead to an increased risk of falls [13, 14]. Furthermore, the cable between the control sensor and stimulator was inconvenient for walking [15].

Instead of a footswitch, Mansfield et al. [16] and Monaghan et al. [17] detected the heel event of the gait cycle in FES-assisted walking using an accelerometer and a uniaxial gyroscope, respectively. The commercially available product WalkAide also uses an accelerometer for this purpose [18]. Electromyography (EMG) signal is also applied as a control source in FES-assisted walking for the detection of volitional intent of muscle [19]. Yeom et al. amplified the EMG signal of the TA and modulated the stimulation intensity in proportion to the integrated EMG envelope. The electrical pulses are then sent to the common peroneal nerve for dropfoot correction [20].

In these studies, FES applied to the TA was mainly triggered by the heel-off event. However, this event occurs during the push-off phase and before TA activation [17]. An earlier start of TA stimulation results in reduced ankle plantarflexion [21]. Spaich et al. suggested implementing a constant time interval before the onset timing of TA stimulation to extend the push-off phase before the ankle dorsiflexion [21]. Some studies have found that walking speed can affect the activation of TA [22, 23]. Shiavi et al. found that the duration of EMG activity decreased as speed increased [22]. In Winter et al.’s study, the shape of the EMG patterns generally remained similar at the different walking speeds and the duration of EMG activity was closely related to the normalized stride time [23]. Although the duration of TA activation changes with the walking speeds has been reported [24], the selection of speed-adaptive FES parameters for TA has not been investigated.

The objective of this study is to find a more physiologically appropriate FES design for dropfoot correction. Firstly, speed-related changes in onset timing and the duration of TA activation were examined. Next, linear models were built for the walking speed and time interval from the heel-off event to the onset timing of TA activation, as well as for the walking speed and the duration of the TA activation. The speed-adaptive stimulation (SAS) timing and duration were then calculated based on the two models and applied for FES-assisted walking. Finally, the performance of stimulation triggered by SAS, heel-off event (HOS) and no stimulation (NS) were compared during FES-assisted walking on both subjects with stroke and healthy subjects at different walking speeds.

## Methods

### Modeling of stimulation parameters based on TA

Nine healthy subjects (five male and four female) were recruited, and none of them had musculoskeletal impairment or injury that affected walking. The mean (±standard deviation (SD)) age of the volunteers was 22.7 (±1.3) years. Two different sensors were attached to the healthy subjects. A footswitch (Tekscan Inc., Boston, MA, USA) was placed on the hindfoot using medical tape to record heel-off and heel-strike events. Circular silver-silver chloride (Ag-AgCl) electrodes (M2223, 3 M Inc., USA) with a diameter of 5 mm and inter-electrode distance of 20 mm were attached to the skin surface of the TA with one reference electrode near the lateral epicondyle of the femur [25].

The surface electromyography signal from the TA was recorded and amplified at a gain of 4000 using a tele-EMG system (MyoSystem2400, Noraxon, USA). Labview software (Labview 2010, National Instruments Corporation, Austin, Texas, USA) stored the footswitch and EMG signals synchronously on a hard disk for offline processing. The signals were obtained using an A-D converter (PCIe-6341, National Instrument, Texas, USA) with 16-bit resolution.

The sampling rate of the footswitch and EMG signals were 100 Hz and 1000 Hz, respectively. The raw EMG signal was first filtered with a bandwidth of 10–150 Hz to remove the low-frequency drift and high-frequency noise [26]. The time interval of TA stimulation was defined as the time span from the heel-off event to the onset timing of TA activation. The timing was defined when the full-wave rectified EMG signal exceeded three times the SD of baseline activity [27]. The baseline activity was the average amplitude of the full-wave rectified EMG signal at rest. The EMG duration was the length of the TA contraction time from the onset timing to the offset timing. The offset timing of TA activation was detected when the rectified signal was less than three times SD of baseline activity [27].

During the experiment, the healthy subjects were instructed to walk on a treadmill (BH, G6425-F3, Spain). A total of eleven speeds were selected for each subject, which ranged from 0.5 to 1.5 m/s with intervals of 0.1 m/s [24]. In each trial, subjects were asked to walk at a certain speed for at least two minutes. The order of the gait speeds was randomly arranged, and a 2-min rest was given between each two trials to avoid muscle fatigue.

Subjects used their preferred stride length during the experiment. To exclude the influence of acceleration and deceleration during treadmill walking, more than 20 steps were averaged for each subject at each speed [28]. After correlation analysis and least square curve fitting, the linear models were built for the walking speed with respect to the time interval from the heel-off event to the onset timing of TA activation, as well as for the EMG duration.

### Speed-adaptive stimulation

**Fig. 1:**
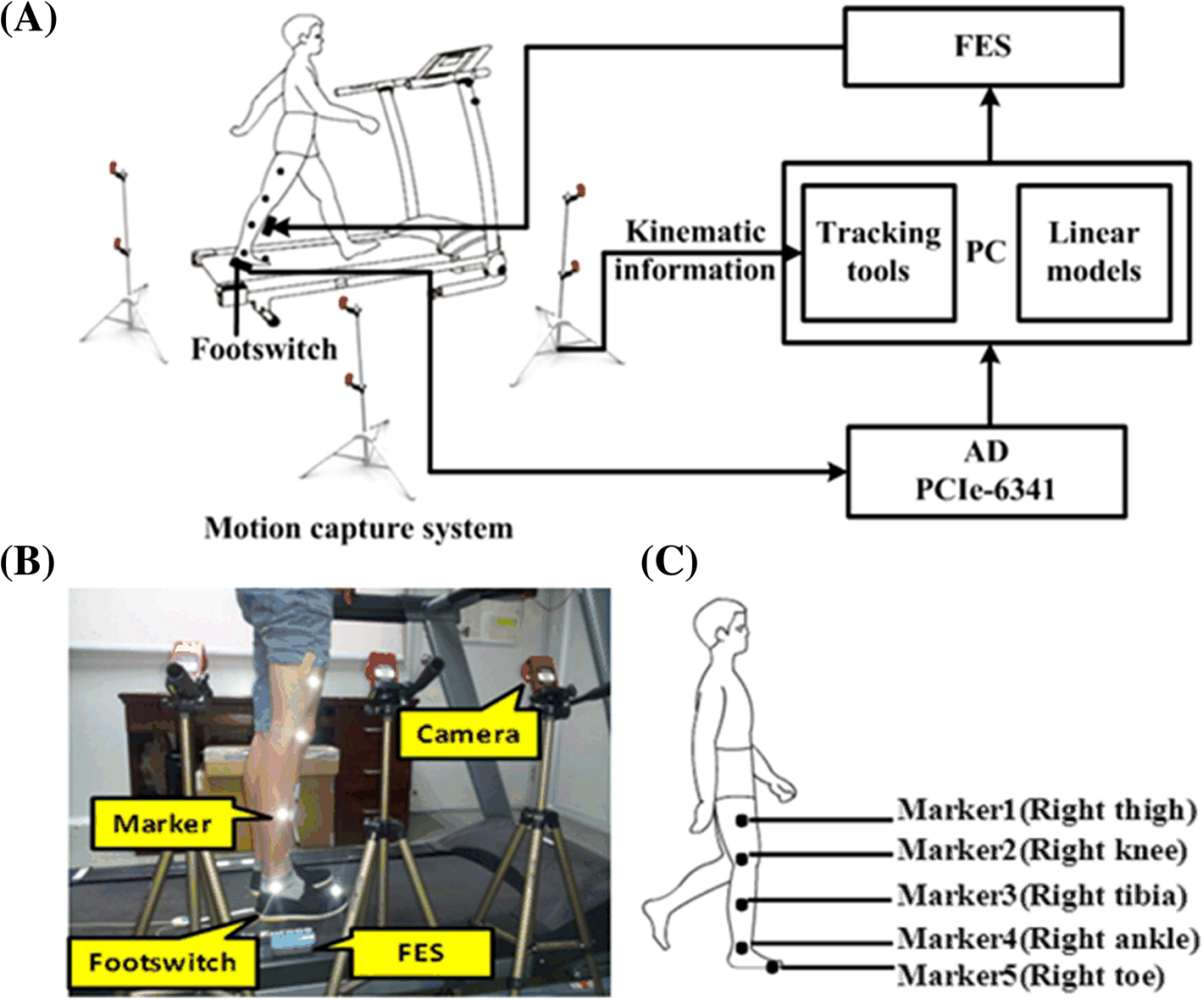
**a** The experiment setup of SAS condition; **b** one healthy subject on the treadmill for system evaluation; **c** the position of five markers on the right leg

**Fig. 2:**
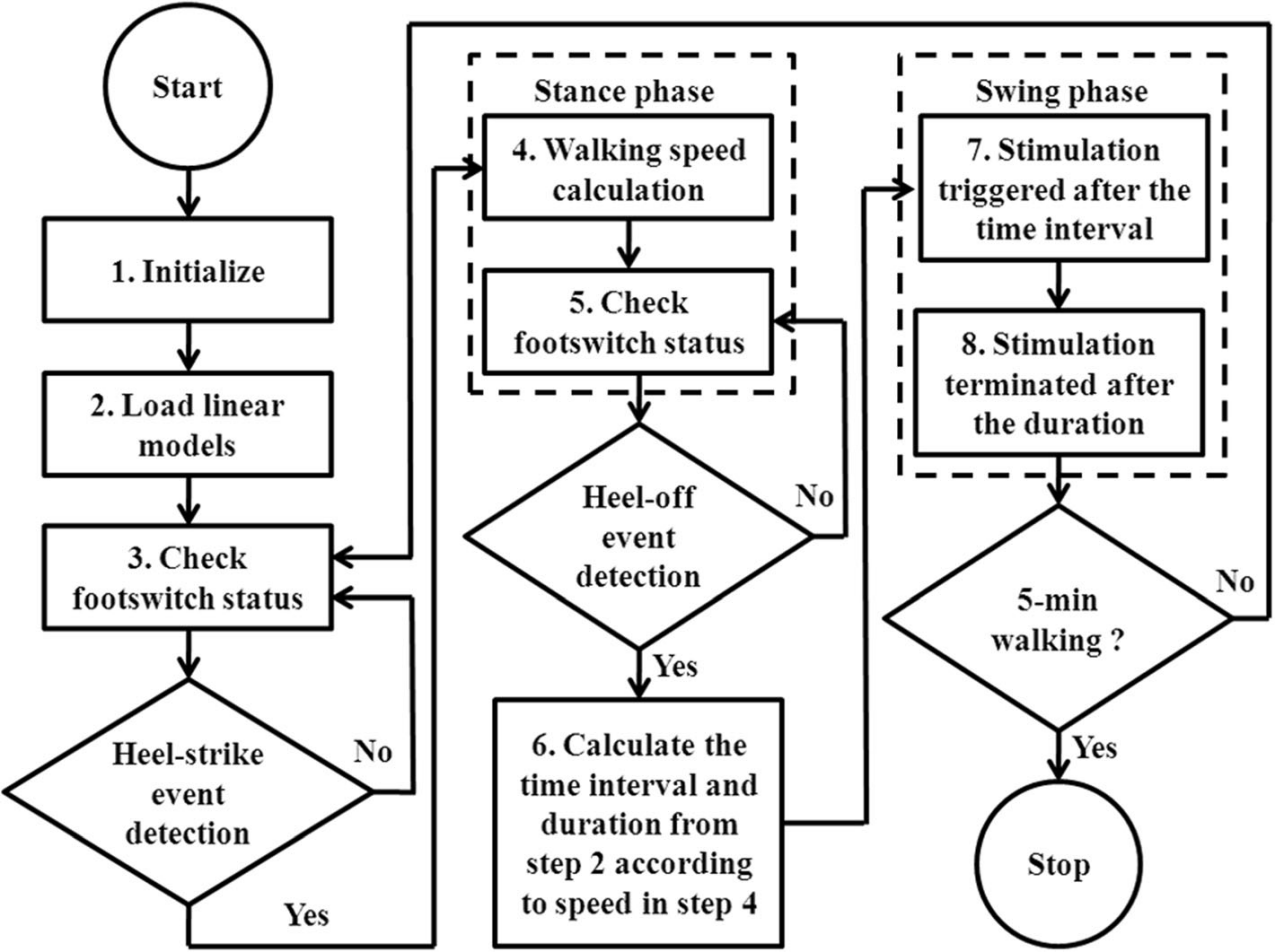
Working flowchart of the SAS control system

The speed-adaptive stimulation is presented in Fig. 1a. A total of five spherical 12-mm reflective markers were attached to the subject’s lower limb (Fig. 1c). Five markers from top to bottom were placed on the following anatomical reference locations: the mid thigh sufficiently distal to the hip, lateral knee joint, the mid shank sufficiently distal to the knee joint, lateral malleolus, and the space between the second and third metatarsal heads [29]. A motion capture system (OptiTrack, NaturalPoint, USA) consisting of six infrared cameras was used to detect the markers at a sampling rate of 100 Hz. The coordinates of each marker were recorded using Tracking Tools software (NaturalPoint, USA) to calculate the walking speed and for further kinematic analysis. The footswitch signal was recorded by the A-D converter and stored in a PC for heel event detection. When the heel-strike event was detected, the walking speed was calculated from the kinematic data of the lower limb marker. The average walking speed of the previous five steps was considered as the current walking speed [25]. After the heel-off event, the time interval before onset timing and duration of TA stimulation were defined from the linear models according to the walking speed. After the speed-adaptive time interval, TA stimulation was triggered. After the speed-adaptive duration, TA stimulation was terminated. The time interval and duration of TA stimlation can be changed according to the different walking speeds. If the walking speeds were lower, the time interval and duration would be longer and if the walking speeds were higher, the time interval and duration would be shorter. A flowchart of the SAS control system is shown in Fig. 2.

### Participants

Nine healthy subjects from the previous experiment and ten subjects with stroke (nine male and one female) with dropfoot (Table 1) took part in the study for system evaluation. The subjects with stroke had sustained a single stroke within at least 6 months prior to study participation and were able to walk on a treadmill independently at multiple speeds without any help. For safety, the subjects with stroke held on to a front handrail during walking [6]. Before participating in the experiment, written informed consent was collected from all the subjects. This study was approved by the ethics committee of the Guangdong Work Injury Rehabilitation Center.

**Table 1:** Demographic and clinical information about subjects with stroke

No.	Sex	Age	Lesion side	Months after stroke	FMA-L	Slow speed (km/h)	Free speed (km/h)	Fast speed (km/h)
1	M	44	L	20	20/34	0.9	1.2	1.5
2	M	38	L	8	25/34	1.0	1.3	1.6
3	F	47	R	15	24/34	1.3	1.7	2.1
4	M	52	R	12	22/34	1.0	1.2	1.4
5	M	51	L	24	20/34	1.0	1.3	1.6
6	M	45	R	12	23/34	1.0	1.2	1.4
7	M	65	L	7	31/34	1.0	1.3	1.6
8	M	41	L	12	23/34	1.0	1.3	1.6
9	M	53	R	12	26/34	1.0	1.3	1.6
10	M	48	L	12	25/34	1.0	1.3	1.6

*M* male, *F* female, *L* left, *R* right, *FMA-L* Fugl-meyer motor assessment for lower limb

### Experiment setup

Healthy subjects were instructed to walk on a treadmill at four speeds: 0.3, 0.6, 0.9, and 1.2 m/s. The subjects walked in four conditions at each speed: FES-assisted walking triggered by the heel-off event (HOS), FES-assisted walking triggered by SAS, and no-stimulation walking (NS). Subjects with stroke were instructed to walk at three speeds: slow, free, and fast. The free speed was the comfortable walking speed on the treadmill, while the fast speed was the maximal tolerable speed (approximately 25 to 30% larger than the free walking speed) [30]. The slow speed was smaller than the free speed with the same proportion. The healthy and subjects with stroke walked in the three conditions at each speed: FES-assisted walking triggered by the heel-off event (HOS), FES-assisted walking triggered by SAS, and no-stimulation walking (NS). Each subject with stroke should finish nine trials and each healthy subject should finish twelve trials. The order of the trials was randomly arranged for both healthy subjects and subjects with stroke. In each trial, the subjects were required to walk for not more than five minutes at a certain speed. A 2-min rest was given between each two trials to avoid muscle fatigue. During the experiment, a footswitch placed on the hindfoot and five reflective makers attached to the lower limb (Fig. 1b) were used for heel event detection and kinematics acquisition.

**Fig. 3:**
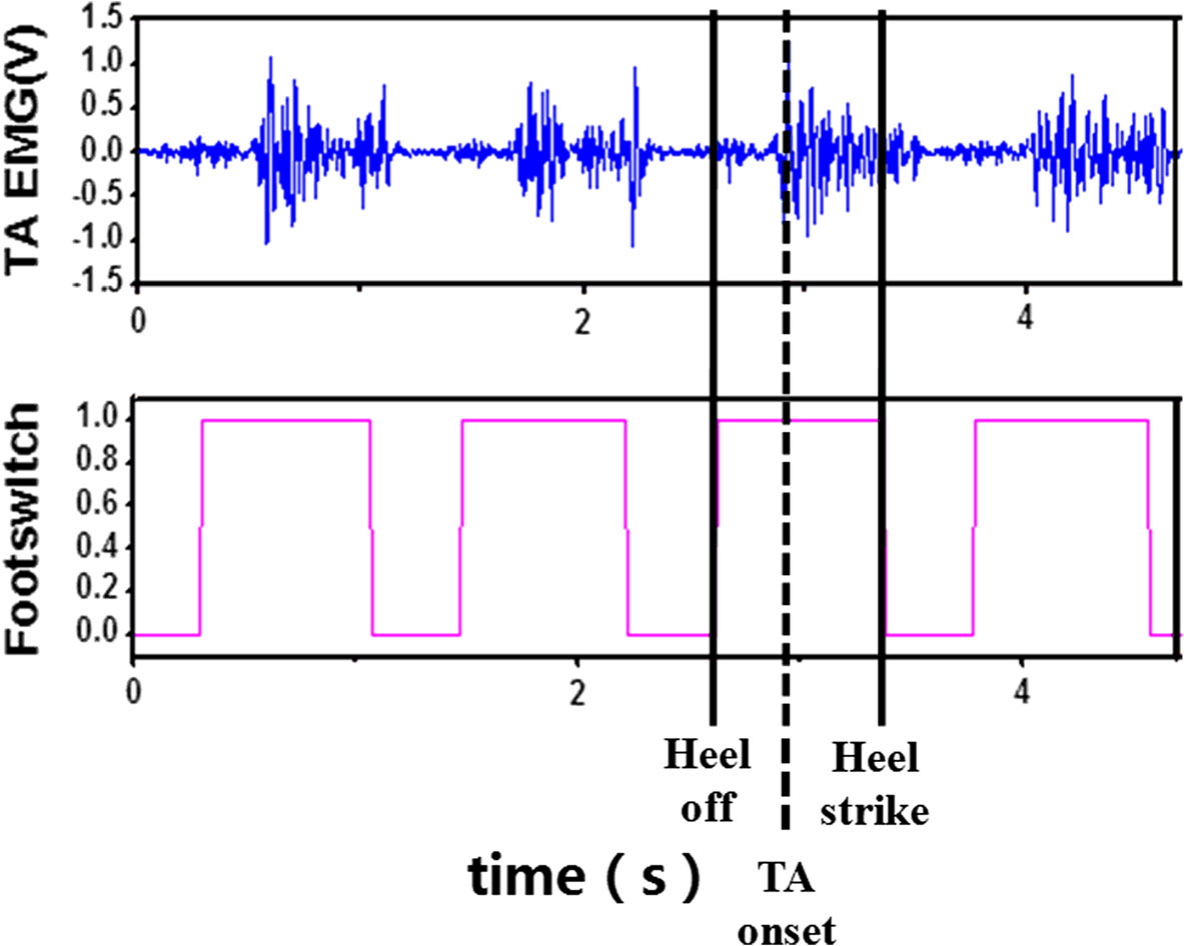
The EMG signal of TA after amplification at a gain of 4000 and the footswitch signals at the speed of 1 m/s of one healthy subject

**Fig. 4:**
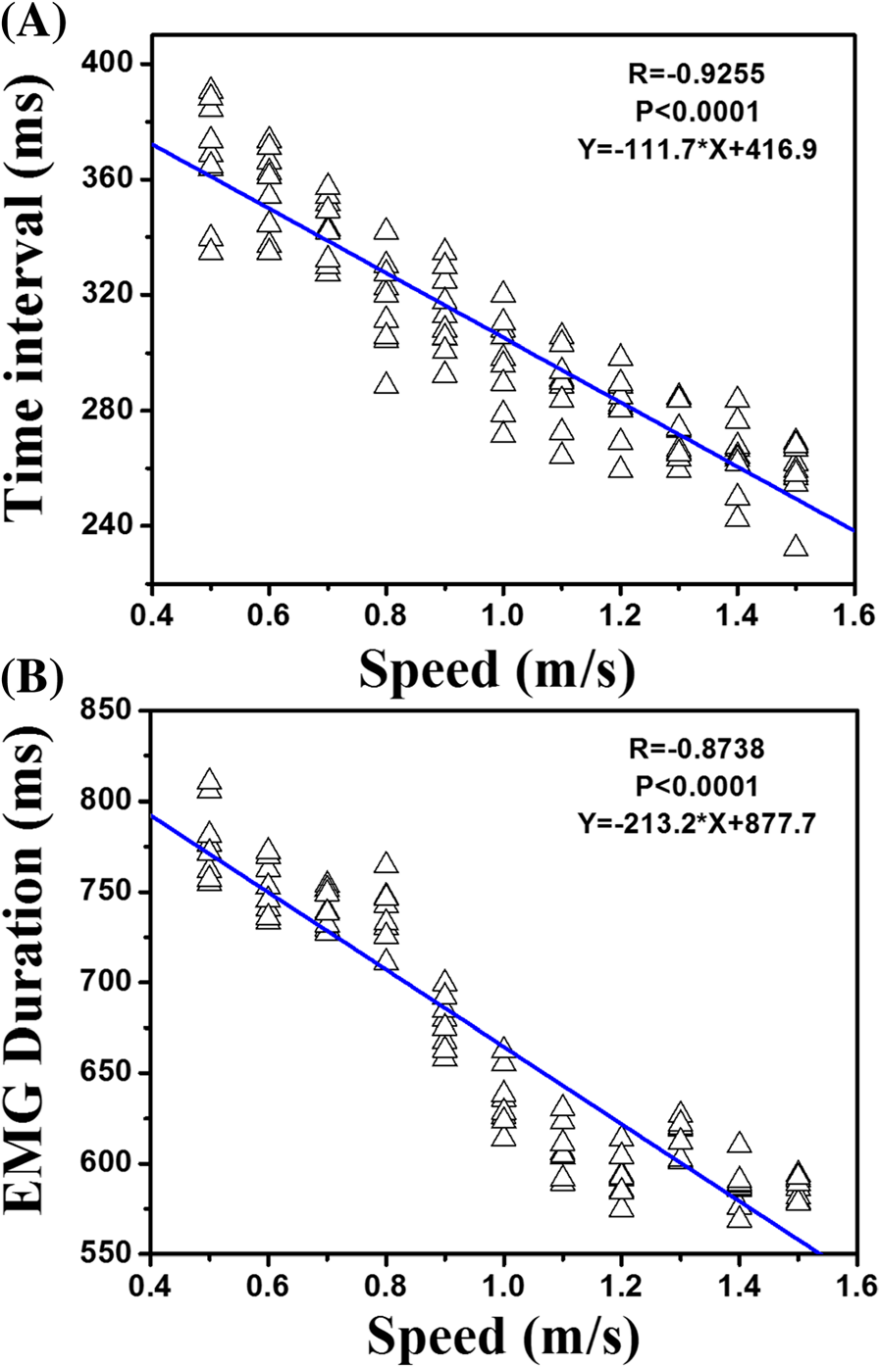
Linear models of walking speed with time interval and EMG duration. R: negative linear correlation; P: significantly linear correlation; The equation is the negative linear model established

In the HOS condition, TA stimulation was triggered and terminated by the heel-off and heel-strike events, respectively [31], the TA stimulation was ahead of the TA activation, and the stimulation duration was shorter than the TA duration in healthy subjects (Fig. 3). In the SAS condition, the onset timing and duration of TA stimulation were defined from the linear models according to the walking speed (Fig. 4), the onset timing and duration of the TA stimulation were in agreement with the onset timing of the TA activation and the TA duration. In the HOS and SAS conditions, the stimulation intensity was set when the subjects achieved a neutral ankle angle (0 degrees) in a seated position with the foot hanging freely in a plantar-flexed position [32]. A functional electrical stimulator was selected (P2–9632, Faisco, China), and the stimulation frequency and the pulse width were 40 Hz and 400 μs, respectively [33]. The shape of the stimulation burst is rectangular, and the stimulation amplitude of the stimulator ranged from 0 to 120 mA.

### Data analysis

The peak knee flexion angle, maximum ankle dorsiflexion angle during the swing phase, and ankle angle at the toe-off event were extracted from kinematic data of the lower limb. A second-order low-pass Butterworth filter was used to filter the kinematic signal with a cutoff frequency of 15 Hz, since a majority of the power was less than 15 Hz according to power spectral analysis [34].

The toe-off event was determined by marker5 on the toe as in a previous study [35], and the heel-off and heel-strike events were detected by a footswitch under the heel. Kolmogorov-Smirnov test was used to assess all the variables for the normality of distribution. Over six strides were averaged for the subjects with stroke in this study [6]. To analyze the influence of the stimulation condition (NS, HOS, and SAS) on ankle dorsiflexion, ankle plantarflexion, and knee flexion angles, one-way analysis of variance with repeated measures was applied to compare the angle values among different conditions. *P* > 0.05 corresponded to the null hypothesis of no significant effect. If there was a significant difference, post-hoc analysis was then carried out using the Bonferroni between different conditions [32]. In each condition, one-way analysis of variance was applied to compare the angle values between the healthy subjects and the subjects with stroke. All statistical analyses were performed using SPSS 19 (SPSS Inc., Chicago, USA), and the level of significance was set at 0.05.

## Results

The Kolmogorov-Smirnov test was applied to the variables, and the results indicated that all the variables followed Gaussian distribution (*P* > 0.05).

### Effect of walking speed on time interval and EMG duration

A graphical representation of the TA activation and footswitch signals is shown in Fig. 3. The first vertical solid line indicates a heel-off event when the footswitch signal is converted from zero to one. The dashed line is the onset timing of TA activation. The time interval is between the first two lines. The second solid line indicates a heel-strike event when the footswitch signals converted from one to zero.

The mean time interval was 367.6 ms in a gait cycle at the slowest speed, which decreased to 258.4 ms at the highest speed. The relationships between the walking speed and time interval are presented in Fig. 4a, and significantly negative linear correlation was found between the walking speed and time interval (*R* = − 0.93, *P* < 0.0001). There was a significant effect of the walking speed on the EMG duration (*P* < 0.01). The mean EMG duration at the speed of 0.5 m/s was 780.0 ms, which decreased by 23.6% to 595.7 ms at the highest speed of 1.5 m/s (Table 2).

**Table 2:** The mean (±S.D.) of time interval and EMG duration at each speed

Speed (m/s)	Time interval (ms)	EMG duration (ms)
0.5	367.63 ± 19.89	780.02 ± 21.23
0.6	356.11 ± 14.41	753.83 ± 15.71^a^
0.7	343.01 ± 11.06^a^	742.82 ± 12.67
0.8	316.90 ± 16.14^a^	741.84 ± 19.75
0.9	314.01 ± 14.03	684.78 ± 25.73^a^
1.0	297.49 ± 15.61^a^	640.95 ± 22.42^a^
1.1	287.96 ± 13.27	611.67 ± 18.06^a^
1.2	281.40 ± 11.48	596.69 ± 16.55
1.3	270.56 ± 8.93	620.92 ± 17.76^a^
1.4	263.89 ± 12.43	593.67 ± 22.95^a^
1.5	258.40 ± 11.18	595.71 ± 27.91

^a^indicated significant difference between the current speed and the previous speed

For most pairs of successive speeds, the change in EMG duration was statistically significant (*P* < 0.05). As shown in Fig. 4b, there was a significantly negative linear correlation between walking speed and EMG duration (*R* = − 0.87, *P* < 0.0001). After the correlation analysis and least square curve fitting, the linear models of walking speed for the time interval and EMG duration were built, as shown in Fig. 4a and b.

### Ankle and knee angle in the three stimulation conditions

**Fig. 5:**
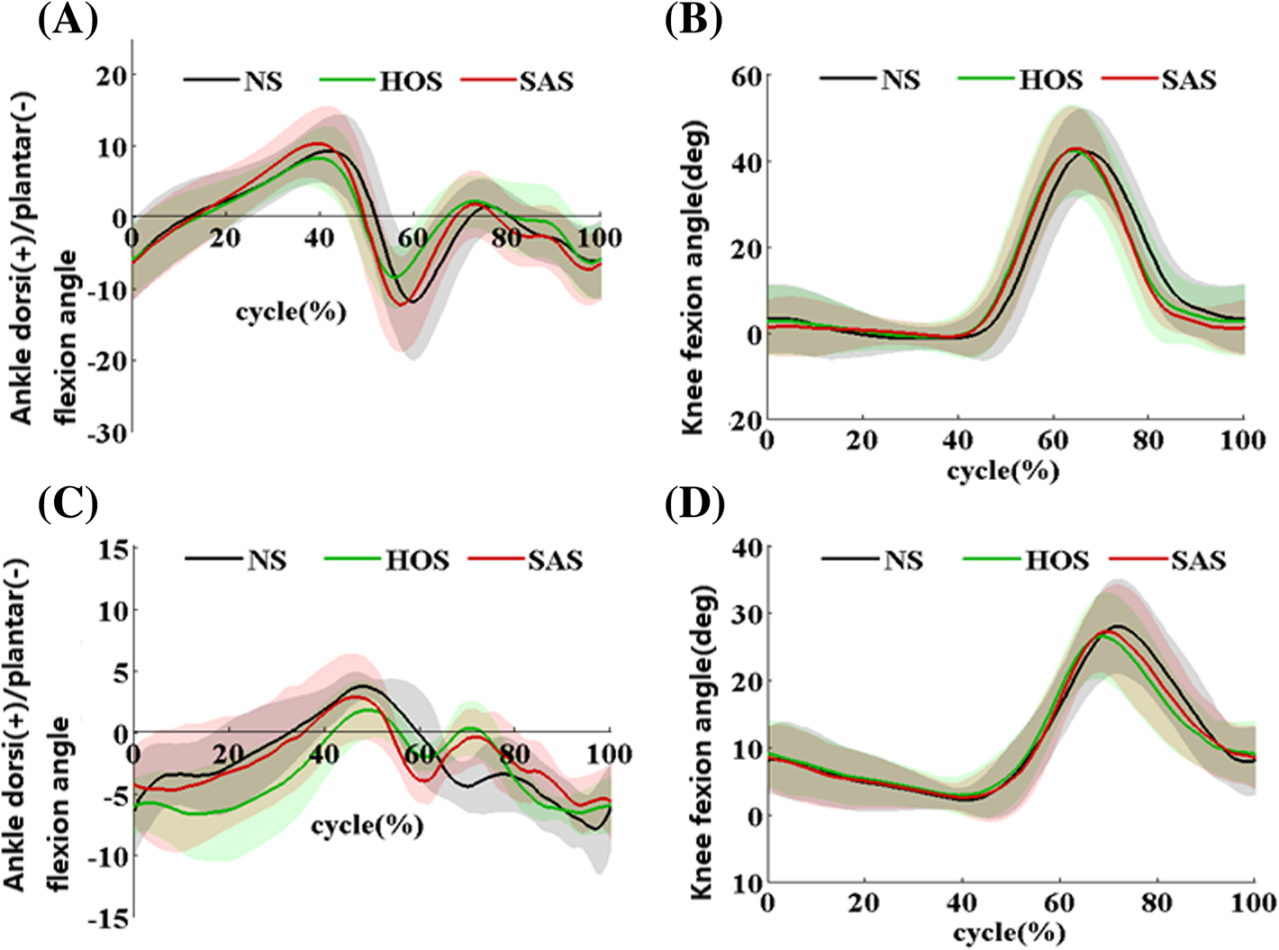
**a** Ankle angles (mean ± std) during the gait cycle for nine healthy subjects at 0.9 m/s; **b** knee angles (mean ± std) during the gait cycle for nine healthy subjects at 0.9 m/s; **c** ankle angles (mean ± std) during the gait cycle for ten post-stroke subjects at free speed; **d** knee angles (mean ± std) during the gait cycle for ten post-stroke subjects at free speed

**Fig. 6:**
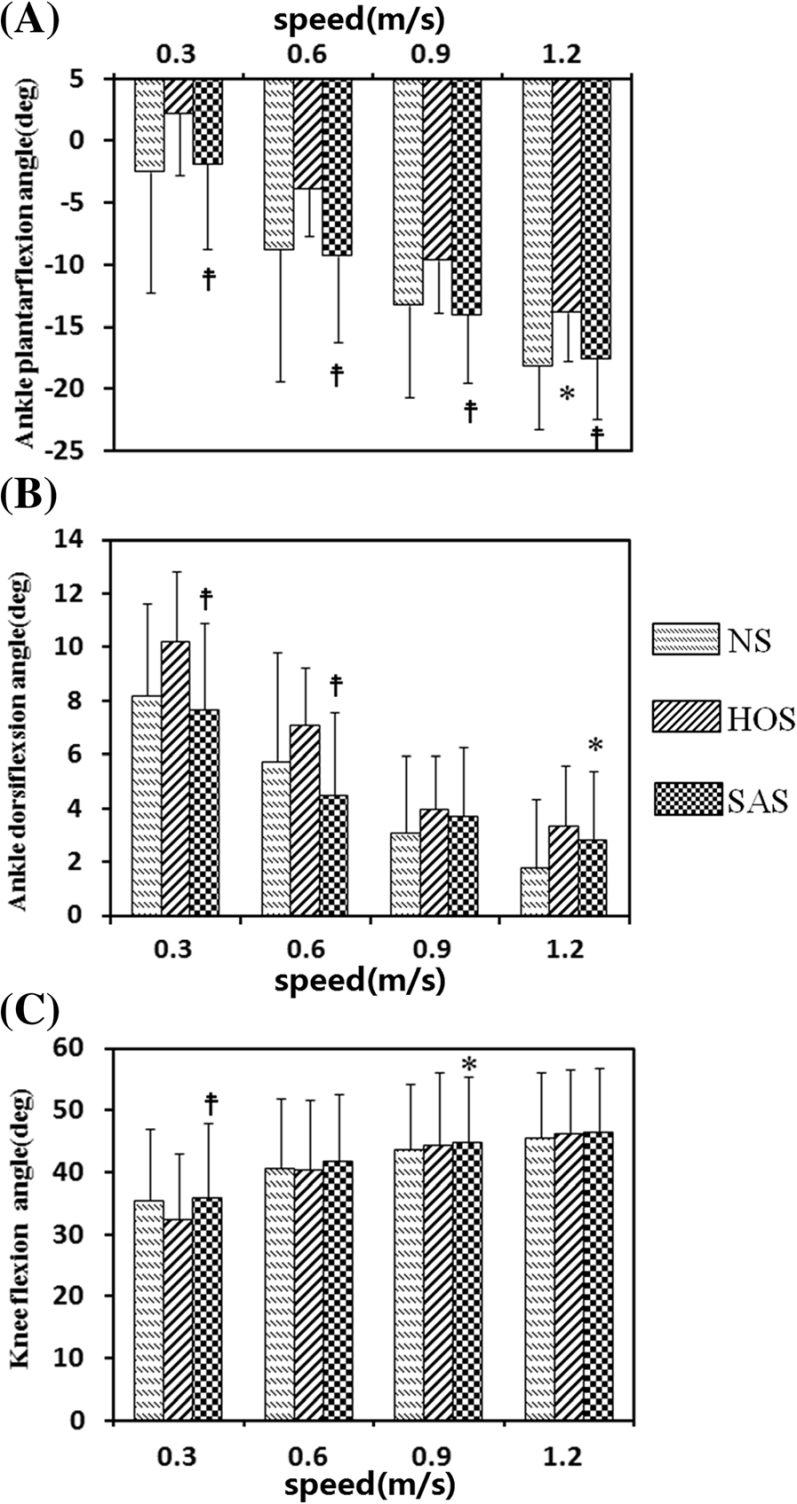
Nine healthy subjects’ results for: **a** Ankle plantarflexion angles at toe-off event; **b** maximum ankle dorsiflexion angles during swing phase; **c** peak knee flexion angles during swing phase; *: significant difference from NS (*P* < 0.05); ☨: significant difference from HOS (*P* < 0.05)

Figure 5a and b present the ankle and knee angles during the gait cycle for nine healthy subjects at 0.9 m/s, respectively. The solid line and the shadow indicate the average angles and the standard deviation during the gait cycle, respectively. Figure 6 presents the maximum ankle dorsiflexion angles during the swing phase, ankle plantarflexion angles at toe-off events, and peak knee flexion angles during the swing phase for the nine healthy subjects. Compared to the NS condition, only the SAS condition achieved larger ankle dorsiflexion angle (2.8 degrees) during the swing phase at 1.2 m/s (*P* < 0.05), but the value SAS condition were significantly smaller compared to that in the HOS condition at 0.3 m/s and 0.6 m/s (*P* < 0.05)

The plantarflexion angle at toe-off events in the HOS condition had the lowest value, which was significantly smaller than the values in the SAS conditions at all speeds (*P* < 0.05) and the value in the NS condition at 1.2 m/s (13.8 degrees versus 18.1 degrees). The plantarflexion angle in the SAS condition was not statistically different from that in the NS condition at all speeds. The peak knee flexion angle in the NS condition was similar to that in the SAS condition at most of speeds, and the peak knee flexion angle in the SAS condition (44.8 degrees) was significantly larger than that in the NS condition (43.7 degrees) at 0.9 m/s (*P* < 0.05). The peak knee flexion angle in the HOS condition had the lowest values at 0.3 m/s and 0.6 m/s, which were significantly lower than that in the SAS condition at 0.3 m/s.

**Fig. 7:**
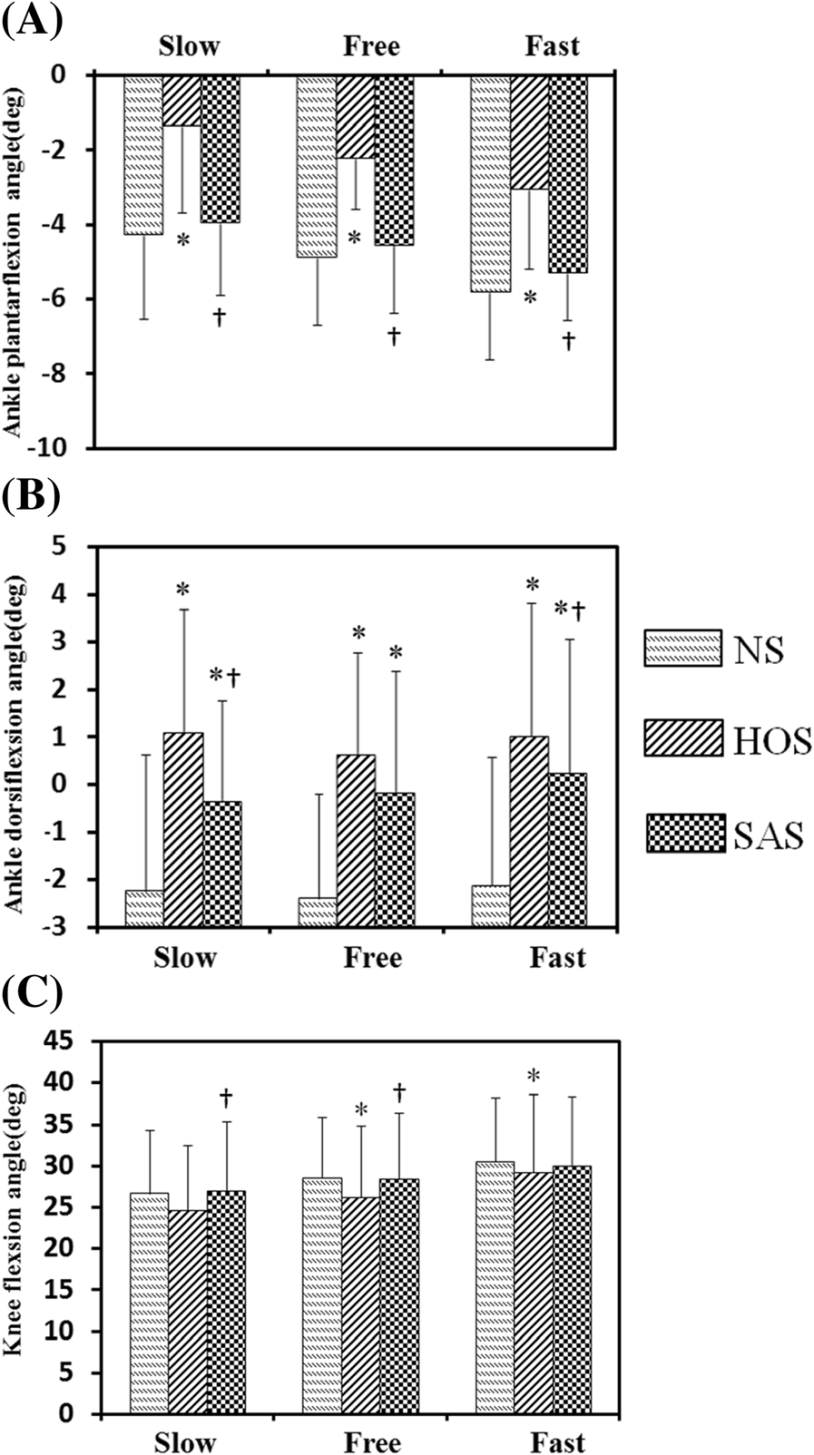
The results of ten subjects with stroke: **a** ankle plantarflexion angles at toe-off event, **b** maximum ankle dorsiflexion angles during swing phase, and **c** peak knee flexion angles during swing phase, *: significant difference from NS (*P* < 0.05), †: significant difference from HOS (*P* < 0.05)

Figure 5c and d present the ankle and the knee angles during the gait cycle for ten subjects with stroke at free speed, respectively. The solid line and the shadow indicate the average angles and the standard deviation during the gait cycle, respectively. Figure 7 presents the maximum ankle dorsiflexion angles during the swing phase, ankle plantarflexion angles at toe-off events, and peak knee flexion angles during the swing phase for ten subjects with stroke. The HOS and SAS conditions achieved larger ankle dorsiflexion angles during the swing phase compared to that in the NS condition, and the values in the SAS conditions were significantly smaller than that in the HOS condition at free speed (*P* < 0.05). At fast speed, the value in the SAS condition (0.23 degrees) was smaller than that in the HOS condition (1.0 degrees). The plantarflexion angles during toe-off events in the HOS condition had the minimum values at all the speeds, which were significantly smaller than that in the NS and SAS conditions. And the plantarflexion angles in the SAS condition were similar with that in the NS condition at all the speeds. The peak knee flexion angles in the SAS condition were significantly larger than that in the HOS condition at slow and free speeds. There were smaller knee flexion angles at free and fast speeds in the HOS condition than that in the NS condition. No significant differences were found in the peak knee flexion angle between the NS and SAS conditions (*P* > 0.05)

**Fig. 8:**
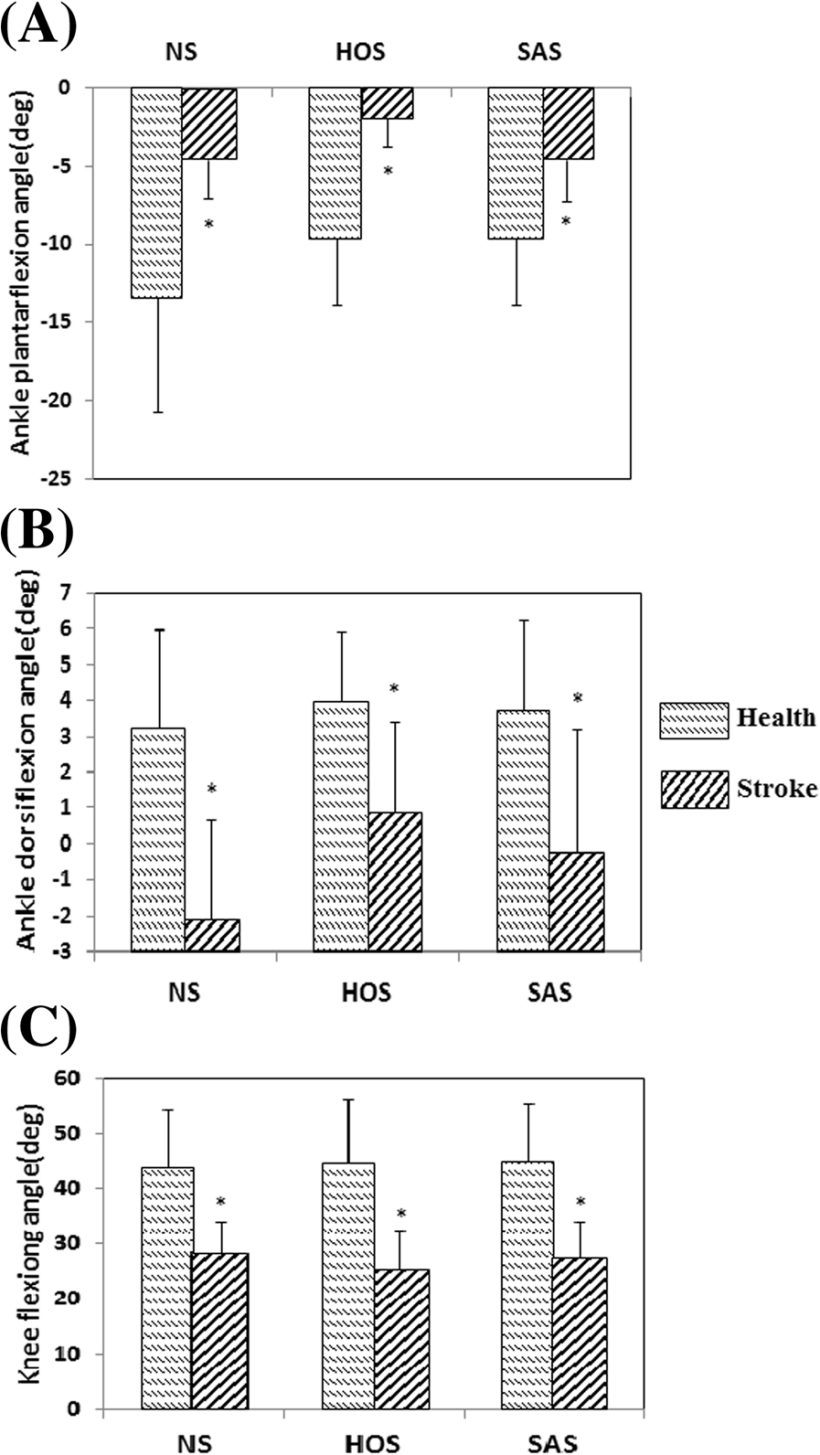
Comparison between healthy subjects and subjects with stroke: **a** Ankle plantarflexion angles at toe-off event; **b** maximum ankle dorsiflexion angles during swing phase; **c** peak knee flexion angles during swing phase; *: *P* < 0.05. The error bars represented the standard deviations

Figure 8 presents the maximum ankle dorsiflexion angles during the swing phase, ankle plantarflexion angles at toe-off events, and peak knee flexion angles during the swing phase for the nine healthy subjects at 0.9 m/s and ten stoke subjects at free speed. In all conditions, healthy subjects achieved larger ankle and knee angles than stoke patients (*P* < 0.05).

## Discussion

FES-assisted ankle dorsiflexion has mainly been triggered by heel-off events in previous studies [7, 33, 36–38]. However, the heel-off event occurred ahead of the actual onset timing of TA activation in healthy subjects (shown in Fig. 3). Therefore, FES-assisted ankle dorsiflexion triggered by the heel-off event may lead to adverse effects, such as reduced ankle plantar flexion during the push-off phase and decreased knee flexion in the swing phase resulted from the decreased forward propulsive force [12]. The stimulation duration to the TA was set to a fixed value for different situations in a previous study, but the physiological relevance of the fixed value was not investigated [8]. If the stimulation duration is shorter than what is needed by the subjects with stroke, a heel-strike event may not emerge due to insufficient ankle dorsiflexion, which might still cause a risk of falls. However, if the stimulation duration is longer than what is needed, the gait may be too unstable for the forefoot to touch the ground immediately after the heel strike, and the subjects would have to decrease their speed to make an adjustment. This might also be adverse for long-term rehabilitation.

Kesar et al. and Springer et al. terminated ankle dorsiflexion stimulation using heel-strike events [32, 37]. As shown in Fig. 3, the TA was still activated at heel-strike events, which suggests that it is not the optimal timing for terminating the dorsiflexion stimulation [32, 37]. Burridge et al. used the Odstock drop foot stimulator (ODFS) for chronic hemiplegic subjects with adjustable extension time after heel strike [39], but they did not mention the selection criteria of stimulation duration [39].

A higher ankle dorsiflexion angle could be observed at all speeds in the subjects with stroke in the HOS and SAS conditions than that in the NS condition. This implies that ankle dorsiflexion can be improved by using FES. An ideal FES intervention for dropfoot correction would be involved increasing ankle dorsiflexion during the swing phase without affecting ankle plantarflexion at toe-off events. However, although the FES produced greater ankle dorsiflexion during the swing phase in previous studies, the ankle plantarflexion at toe-off events was worsened [6, 21, 32], and a similar result could also be found in subjects in the HOS conditions. One of the reasons was that the stimulation timing in the HOS condition was triggered by heel-off events, as in most previous studies [7, 33, 36–38]. However, as shown in Fig. 3, the heel-off event was ahead of the actual onset timing of TA activation.

In the SAS condition, the FES was triggered by heel-off events after a time interval, which was associated with the linear model. Moreover, the duration of the time interval in the SAS condition was speed-adaptive rather than a constant value, as in previous study [8]. This design could account for the effect of walking speed. In Table 2 the standard deviation of time interval and EMG duration was very small, so the model was accurate enough to account for the individual differences. The FES parameter settings of the patients were referred to the EMG data of healthy subjects in previous studies, because FES that mimicked normal TA activation mode can help subjects with stroke recover in a natural way [8, 9, 25]. Therefore, data from healthy young subjects were collected to develop the linear model offline, and the model was applied to control the speed-adaptive onset timing and duration for the FES system in real time. In our study, the ankle plantarflexion angle in the SAS condition was similar to that in no-stimulation walking, and it was significantly larger than that in the HOS condition. This indicates that the speed-adaptive onset timing and duration are physiologically appropriate for dropfoot correction. According to the previous studies, the knee angle plays a critical role in generating the forward propulsion [40, 41]. An improper take-off during the push-off phase is attributed to reduced ankle plantar flexion during the push-off phase and decreased knee flexion in the swing phase resulted from the decreased forward propulsive force [6, 12]**.** Therefore, the larger peak knee flexion angle might be the consequence of the larger reserved forward propulsive force in the SAS condition than that in the HOS condition in this study. We implemented an adaptive time interval before the onset timing of TA stimulation to extend the push-off phase, so the knee angle in SAS condition was similar with that in NS condition and different from that in HOS condition. The plantarflexion angle in the SAS condition more closely approached that in the NS condition and was larger than that in the HOS condition at all the speeds.

## Conclusions

Although FES could correct dropfoot, it may come at the cost of impairing ankle plantarflexion if the ankle dorsiflexion stimulation is not well timed. In this study, the footswitch sensor and the kinematic signal combined with the linear model contributed a correction in stimulation timing in order to improve ankle plantarflexion. It should be noted that the phases of stimulation between HOS and SAS was not evaluated in detail in this study. In future work, it will be further investigated to improve the performance of FES-assisted walking together with many other factors that may also account for the change of ankle dorsiflexion and plantarflexion angles, such as stimulation intensity, step length, swing/stance time. Although the model was developed by the data from healthy young subjects within limited range of walking speeds (0.5 m/s - 1.5 m/s), linear relationships can be extended to a wider range of speeds. Moreover, the speed-adaptive stimulation system could be transferred from treadmill walking to walking over ground. An inertial sensor could be used to replace the motion capture system for the walking speed calculation and the FES controller. The SAS system with the motion tracking system is useful for indoor treadmill-based rehabilitation mainly in hospital. If the inertial sensor is used to estimate the walking speeds in outdoor daily-life walking for subjects with stroke, where acceleration or deceleration exits, the linear model can also apply to calculate the speed adaptive time interval and duration of stimulation.

## Abbreviations

EMG: Electromyography
FES: Fuctional electrical stimulation
HOS: Heel-off event stimulation
NS: No-stimulation walking
ODFS: Odstock drop foot stimulator
SAS: Speed-adaptive stimulation
SCI: Spinal cord injury
TA: Tibialis anterior
